# The Effects of Exercise Under Hypoxia on Cognitive Function

**DOI:** 10.1371/journal.pone.0063630

**Published:** 2013-05-10

**Authors:** Soichi Ando, Yoichi Hatamoto, Mizuki Sudo, Akira Kiyonaga, Hiroaki Tanaka, Yasuki Higaki

**Affiliations:** 1 Faculty of Sports and Health Science, Fukuoka University, Fukuoka, Japan; 2 Graduate School of Sports and Health Science, Fukuoka University, Fukuoka, Japan; 3 Institute for Physical Activity, Fukuoka University, Fukuoka, Japan; University of Medicine & Dentistry of NJ - New Jersey Medical School, United States of America

## Abstract

Increasing evidence suggests that cognitive function improves during a single bout of moderate exercise. In contrast, exercise under hypoxia may compromise the availability of oxygen. Given that brain function and tissue integrity are dependent on a continuous and sufficient oxygen supply, exercise under hypoxia may impair cognitive function. However, it remains unclear how exercise under hypoxia affects cognitive function. The purpose of this study was to examine the effects of exercise under different levels of hypoxia on cognitive function. Twelve participants performed a cognitive task at rest and during exercise at various fractions of inspired oxygen (FIO_2_: 0.209, 0.18, and 0.15). Exercise intensity corresponded to 60% of peak oxygen uptake under normoxia. The participants performed a Go/No-Go task requiring executive control. Cognitive function was evaluated using the speed of response (reaction time) and response accuracy. We monitored pulse oximetric saturation (SpO_2_) and cerebral oxygenation to assess oxygen availability. SpO_2_ and cerebral oxygenation progressively decreased during exercise as the FIO_2_ level decreased. Nevertheless, the reaction time in the Go-trial significantly decreased during moderate exercise. Hypoxia did not affect reaction time. Neither exercise nor difference in FIO_2_ level affected response accuracy. An additional experiment indicated that cognitive function was not altered without exercise. These results suggest that the improvement in cognitive function is attributable to exercise, and that hypoxia has no effects on cognitive function at least under the present experimental condition. Exercise-cognition interaction should be further investigated under various environmental and exercise conditions.

## Introduction

Many sports are performed in a dynamic and ever-changing environment. Players have to make optimal decisions as quickly as possible under conditions of physiological stress. Hence, cognitive function is an important determinant of performance, and high-level cognitive abilities are required during exercise. A number of studies have demonstrated that cognitive function improves during a single bout of moderate exercise [1], [2]. These findings suggest that physiological changes induced by acute exercise have the potential to improve cognitive function. However, the specific mechanisms by which exercise affects cognitive function remain largely unclear.

Recently, Dietrich and Audiffren [3] proposed a reticular-activating hypofrontality model to account for the psychological consequences of acute exercise. According to this model, exercise may facilitate implicit information by enhanced noradrenergic and dopaminergic systems. On the other hand, extensive activation of motor and sensory systems during strenuous exercise may attenuate higher-order functions of the prefrontal cortex because the brain has finite metabolic resources. Therefore, the effects of acute exercise on cognitive function may be determined by the balance between the metabolic demands and beneficial effects of exercise in the brain. The reported improvement in cognitive function during moderate exercise suggests that the beneficial effects predominantly occur during moderate exercise.

Oxygen delivery to the brain tissue may be compromised under hypoxia. Indeed, high-altitude exposure may lead to acute altitude sickness, pulmonary and cerebral edema [4]. Hypoxia is thought to have the detrimental effects on the central nervous system [5]–[7]. Hypoxia can be a cause of neurological and physiological deficits as well as structural damage in the brain tissue, as evidenced by a growing body of literature, including neuroanatomy [8]–[16], neurophysiology [8], [17]–[19], and neuropsychological measures [8], [20], [21]. Accordingly, hypoxia has the potential to impair brain function [22].

It has been suggested that cognitive function may be impaired under hypoxia [23], [24].Hypoxia decreases arterial pressure of O_2_ (PaO_2_) and arterial saturation of O_2_ (SaO_2_) [25], [26]. At the cellular level, the turnover of several neurotransmitters seems to be altered under hypoxia despite the preserved state of brain energy stores [27]. For example, the synthesis of acetylcholine is sensitive to oxygen availability [28]. Thus, brain desaturation and resultant biological process may be responsible for the impairment of cognitive function although the underlying mechanisms are still unclear. Notably, the impairment of cognitive function was prominent at high altitude [23], [24]. As altitude increases and, thus, severity of hypoxia increases, PaO_2_ and SaO_2_ gradually decrease [25], [26]. It is possible that the detrimental effects of hypoxia on cognitive function are exaggerated as the severity of hypoxia increases.

Exercise under hypoxia substantially decreases arterial oxygen saturation and cerebral oxygenation relative to normoxia [29]–[31], which suggests that oxygen availability may be compromised in the brain during exercise under hypoxia. Given that brain function and tissue integrity are dependent on continuous and sufficient oxygen supply, we hypothesized that cognitive function is impaired during exercise under hypoxia as the severity of hypoxia increases. However, it remains unclear how exercise under different levels of hypoxia affects cognitive function. It is important to examine the effects of exercise under different levels of hypoxia on cognitive function to understand how cognitive function is affected during exercise at different levels of altitude.

The purpose of this study was to examine the effects of exercise under different levels of hypoxia on cognitive function. In particular, we focused on whether the improvement in cognitive function during moderate exercise is still present under hypoxia or whether cognitive function is impaired during exercise under hypoxia as the severity of hypoxia increases. We also investigated whether there was an association between cognitive function and oxygen availability during exercise under hypoxia. Our findings will provide important information about exercise-cognition interaction under conditions of physiological stress at different levels of altitude.

## Materials and Methods

### Ethics Statement

This study was approved by the ethics committee of Fukuoka University and was in accordance with the Declaration of Helsinki. All participants gave written informed consent to participation.

### Participants

Twelve male participants (mean ± SD, age = 22.9±1.5 yr; height = 1.71±0.06 m; body mass = 64.7±7.3 kg) were recruited to participate in this study. The participants were not currently engaged in regular training. However, they were physically active and did not have any history of cardiovascular, cerebrovascular, or respiratory disease. Participants were asked to refrain from strenuous exercise for at least 48 h before each experiment.

### Cognitive Task

**Figure 1:**
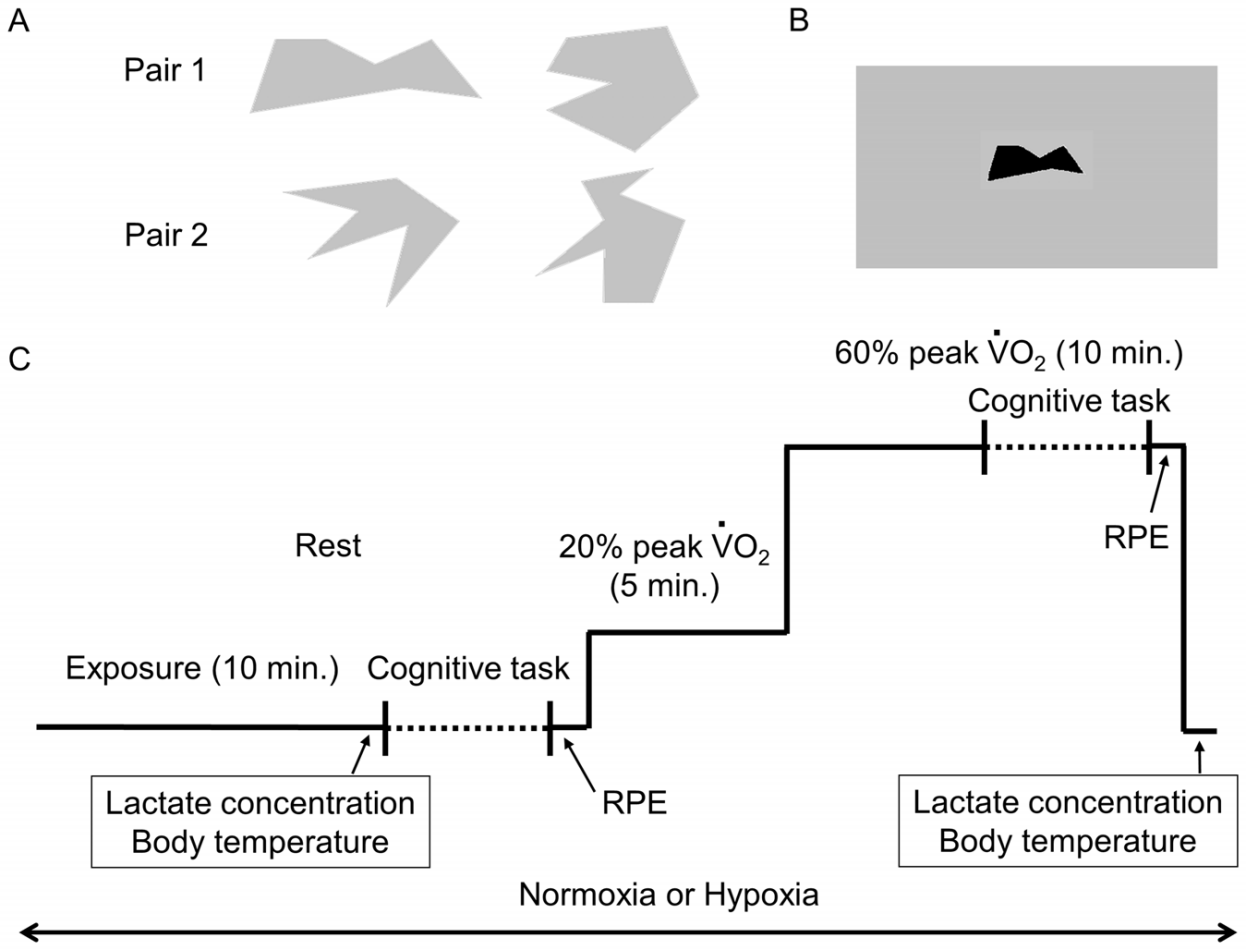
Illustration of figures and experimental protocol. (A) Examples of the paired figures. (B) Presentation of the figure. (C) Illustration of the experimental protocol. The dashed lines show the duration of the cognitive task. The arrows indicate the times at which blood lactate concentration, body temperature, and RPE were measured.

Participants performed a modified version of the Go/No-Go task [32]. We used a laptop computer (Let’s note CF-R4, Panasonic, Osaka, Japan) to control visual stimulus presentation and record reaction times. During the cognitive task, participants were seated on a cycle ergometer, facing a 17-inch computer display at a viewing distance of 80 cm. At the beginning of the cognitive task, an instruction to press a computer mouse button appeared on the screen. The computer mouse was horizontally situated above the right handlebar, so that participants were able to respond with the right finger while cycling. The cognitive task was started by pressing the mouse button with the right middle finger. One of the paired figures (Fig. 1*A*) was then presented in blue at the center of the screen (Fig. 1*B*). In the case of a Go-trial, participants released the mouse button as quickly as possible, and a correct or incorrect feedback tone was presented for 0.5 s. In the case of No-Go trials, participants continued holding the mouse button, and the feedback tone was presented for 0.5 s. After feedback, the trial was reset and instructions about the next trial were presented on the screen. Once participants completed five successive correct Go and No-Go trials, the relationship between correct response and figure was reversed. After the next five successive correct responses, the new paired figures were presented. The participants completed the five successive correct responses six times. The number of trials in the cognitive task was 38.2±5.7 trials, which was dependent on the number of errors the participants made. The number of trials was not different among environmental conditions. The average time taken to complete the cognitive task was 187±25 s. We averaged reaction times in the Go-trials for each participant. Reaction time was not measured in the No-Go trials. Response accuracy was calculated as the number of correct trials divided by the total number of the trials. For this calculation, we excluded trials immediately after the relationship between correct response and figure was reversed or one of the new paired figures was presented.

### Experimental Procedure

The experiment was performed on four non-consecutive days. On the first day, participants performed a maximal exercise test on a cycle ergometer (75XLII, COMBI Wellness, Tokyo, Japan) to determine their peak VO_2_. Following a warm-up exercise at 10 W for 4 min, a maximal exercise test was initiated at a freely chosen pace with 15 W increments every minute in a step-wise manner. The maximal exercise test was stopped when participants reached their limit of tolerance. Ventilatory parameters were measured using a gas analysis system (ARCO-2000, ARCO System, Chiba, Japan). The peak VO_2_ was taken as the highest VO_2_ attained. The mean peak VO_2_ was 54.0±7.3 ml • kg^−1^ • min^−1^.

On the days of the main experiments, participants performed cognitive tasks under either normoxia [fraction of inspired oxygen (FIO_2_) = 0.209] or normobaric hypoxia (FIO_2_ = 0.18 and 0.15). FIO_2_ values of 0.18 and 0.15 correspond to altitudes of approximately 1,300 m and 2,600 m, respectively. All experiments were conducted in an environmental control chamber (FHC-20S, Fuji-ika Sangyo, Chiba, Japan). The ambient temperature was maintained at 22°C and the relative humidity was controlled at 50%. The order of normoxic and hypoxic conditions was randomized across participants, who were blinded to the respective conditions. A few days before the first main experiment, participants completed several practice blocks of the cognitive tasks while sitting on the cycle ergometer. These practice blocks minimized the possibility that learning effects could interfere with the effects of acute physiological changes.

Fig. 1*C* summarizes the experimental protocol. At the beginning of the main experiment, participants were exposed to either normoxia or hypoxia for 10 min. The participants were exposed to normoxia as well as hypoxia because they were unaware of the experimental conditions. Then, participants performed the cognitive task while sitting on the ergometer under the respective condition. After the cognitive task at rest, the participants cycled on the ergometer at 20% peak VO_2_ (46.8±9.1 W) for 5 min as a warm-up exercise. They then cycled at 60% peak VO_2_ (158.2±20.9 W) for 10 min. Exercise was also performed under the respective condition. Cognitive function was assessed during exercise at 60% peak VO_2_ because our previous study has shown improvement in cognitive function at this intensity [33]. Cognitive function was measured 5 min after the start of exercise at 60% peak VO_2_. The pedaling rate was freely chosen by each participant.

We performed an additional experiment to confirm that changes in reaction time were not caused by learning effects arising from repetition of the tasks. This experiment also confirmed the effects of exercise on cognitive function. Eight participants took part in the additional experiment. Two participants started with this experiment, and then they performed the other conditions in a randomized order on different days. In the additional experiment, they performed cognitive tasks while sitting on the ergometer without exercise. Cognitive tasks were performed twice (first and second cognitive tasks) in the same time course (see Fig. 1*C*) under normoxia in the environmental control chamber.

### Physiological Parameter Measurements

Heart rate (HR) was measured from the R-R interval of an electrocardiogram (ECG) using a three-lead system. Pulse oximetric saturation (SpO_2_) was monitored using a pulse oximeter (OLV-3100, Nihon Kohden, Tokyo, Japan) placed on the left index finger. We recorded ratings of perceived exertion (RPE; 6–20 Borg scale) [34] immediately after each cognitive task. Perception of exertion reflects integration of a variety of perceptual cues including afferent feedback from cardiovascular and pulmonary systems [35]. In the present study, we measured RPE to investigate whether perception of exertion is associated with an altered cognitive function. Blood lactate concentration and body temperature were measured at rest and immediately after exercise. Capillary blood was collected from the right earlobe, and the blood lactate concentration was determined with the lactate oxidase method using an automated analyzer (Lactate Pro, Arkray, Kyoto, Japan). Body temperature was measured from the tympanic membrane.

### Near-infrared Spectroscopy (NIRS) Measurement

Most biological tissue is relatively transparent to near-infrared light between apparently 700–1000 nm because water absorption and hemoglobin absorption are relatively small within this wavelength region [36]. Once the brain tissue is irradiated with near-infrared light, the light propagates through the brain tissue. Propagation of near-infrared light through the brain tissue is dependent on absorption and scattering. Near-infrared light is thought to travel through a banana-shaped trajectory from a light source probe to a detector, penetrating the surface of the cortex [37]. The modified Beer-Lambert law, which provides the physical and mathematical basis for NIRS, enables us to continuously measure concentration changes in oxyhemoglobin (oxy-Hb) and deoxyhemoglobin (deoxy-Hb) by assuming that scattering is constant during the measurement [36]–[38]. Total hemoglobin (total-Hb) is calculated as the sum of oxy-Hb and deoxy-Hb. Cerebral oxygenation was expressed as oxy-Hb/total-Hb×100 (i.e. as a percentage). Despite several limitations [31], [37], [39], NIRS allows the measurement of hemoglobin concentrations and cerebral oxygenation qualitatively during exercise in a non-invasive manner.

In, the present study, we used a NIRS system (BOM-L1 TRW, Omegawave, Tokyo, Japan) to monitor the hemoglobin concentrations and cerebral oxygenation. The analog outputs were digitized using a Powerlab analog-to-digital converter (ML795 Powerlab/16sp, A/D Instruments Japan). A probe holder was attached at the left side of the forehead, and a black cloth was wrapped around the probe holder to shield it from ambient light. The probe holder contained one light source probe and two detectors placed at 2 cm (detector 1) and 4 cm (detector 2) from the source. The source generated three wavelengths of near-infrared light (780, 810, and 830 nm). The actual length of the path which near-infrared light travels is several times the distance between the light source probe and the detector. Hence, hemoglobin concentrations were corrected by an age-dependent differential path-lengthfactor [40]. The hemoglobin concentrations received by detector 1 were then subtracted from those received by detector 2 to minimize the effects of near-surface blood flow (*e.g.*, skin blood flow) on hemoglobin concentrations in the cortical tissue [30], [33]. Cerebral oxygenation was expressed as oxy-Hb/total-Hb×100 (i.e. as a percentage).

Before the cognitive task at rest, we measured hemoglobin concentrations for 30 s as a baseline while participants were at rest on the ergometer. Hemoglobin concentrations and cerebral oxygenation during the cognitive tasks was expressed relative to the baseline under normoxia. We used the nasion, eyebrow, and hairline as landmarks on the forehead to make sure that the probe holder was set at the same position on different experimental days [30].

### Data and Statistical Analysis

HR, SpO_2_, cerebral oxygenation, hemoglobin concentrations were averaged during the cognitive tasks at rest and during exercise. We used two-way repeated-measures ANOVA with FIO_2_ level (0.209, 0.18, or 0.15) and exercise (rest or exercise) as the within-subjects factors for each dependent variable. The degree of freedom was corrected using the Huynh Feldt Epsilon when the assumption of sphericity was violated. Tukey’s *post hoc* test or paired *t*-tests were conducted to compare differences where appropriate. All data are expressed as mean ± SD. The significance level was set at *P*<0.05.

## Results

### Physiological Parameters, SpO_2_, Cerebral Oxygenation, and Hemoglobin Concentrations

Table 1 summarizes the results of physiological parameters. There was a significant interaction on HR between FIO_2_ level and exercise [F(2,22) = 11.47, *P*<0.001]. The main effects of FIO_2_ level [F(1.46,16.03) = 5.70, *P*<0.05] and exercise [F(1,11) = 609.18, *P*<0.001] were also significant. By the same token, there was a significant interaction on RPE between FIO_2_ level and exercise [F(2,22) = 13.82, *P*<0.001]. The main effects of FIO_2_ level [F(2,22) = 9.22, *P*<0.01] and exercise [F(1,11) = 490.64, *P*<0.001] were significant. We observed significant increases in HR and RPE during exercise in all FIO_2_ conditions (*P*<0.001, respectively). HR was higher during exercise under hypoxia at 15% O_2_ compared with normoxia and hypoxia at 18% O_2_ (*P*<0.001, respectively). RPE was greater during exercise under hypoxia at 15% O_2_ compared with normoxia (*P*<0.001) and hypoxia at 18% O_2_ (*P*<0.01).

**Table 1:** HR, blood Lactate, RPE, and body temperature under normoxia, hypoxia at 18% O_2_, and hypoxia at 15% O_2_.

Condition	Variables	Rest	Exercise	After
Normoxia				
	HR, bpm	70.3±7.7	158.9±14.3^a^	–
	RPE	6.2±0.4	15.3±1.2^a^	–
	Blood lactate,mmol/l	1.2±0.2	–	5.3±1.5^a^
	Body temperature,°C	36.7±0.3	–	37.1±0.4^a^
Hypoxia (18%)				
	HR, bpm	71.1±7.8	160.7±14.4^a^	–
	RPE	6.3±0.6	15.3±1.7^a^	–
	Blood lactate,mmol/l	1.3±0.4	–	5.7±1.5^a^
	Body temperature,°C	36.6±0.4	–	37.0±0.5^a^
Hypoxia (15%)				
	HR, bpm	71.0±7.1	169.6±14.1a,b,d	–
	RPE	6.1±0.3	16.8±1.7a,b,c	–
	Blood lactate,mmol/l	1.2±0.4	–	7.2±2.0a,b,c
	Body temperature,°C	36.5±0.3	–	36.9±0.4^a^

Values are mean ± SD.

a p<0.001 vs. Rest;

b p<0.001 vs. Normoxia;

c p<0.01,

d p<0.001 vs. Hypoxia at 18% O_2_.

We observed a significant interaction on blood lactate between FIO_2_ level and exercise [F(2,22) = 12.56, *P*<0.001]. There were significant main effects of FIO_2_ level [F(2,22) = 12.82, *P*<0.001] and exercise [F(1,11) = 124.34, *P*<0.001]. Blood lactate increased after exercise in all FIO_2_ conditions (*P*<0.001, respectively). Blood lactate concentration after exercise under hypoxia at 15% O_2_ was greater compared with normoxia (*P*<0.001) and hypoxia at 18% O_2_ (*P*<0.01). In addition, we found a significant main effect of exercise on body temperature [F(1,11) = 37.00, *P*<0.001], indicating that body temperature increased after exercise.

**Figure 2:**
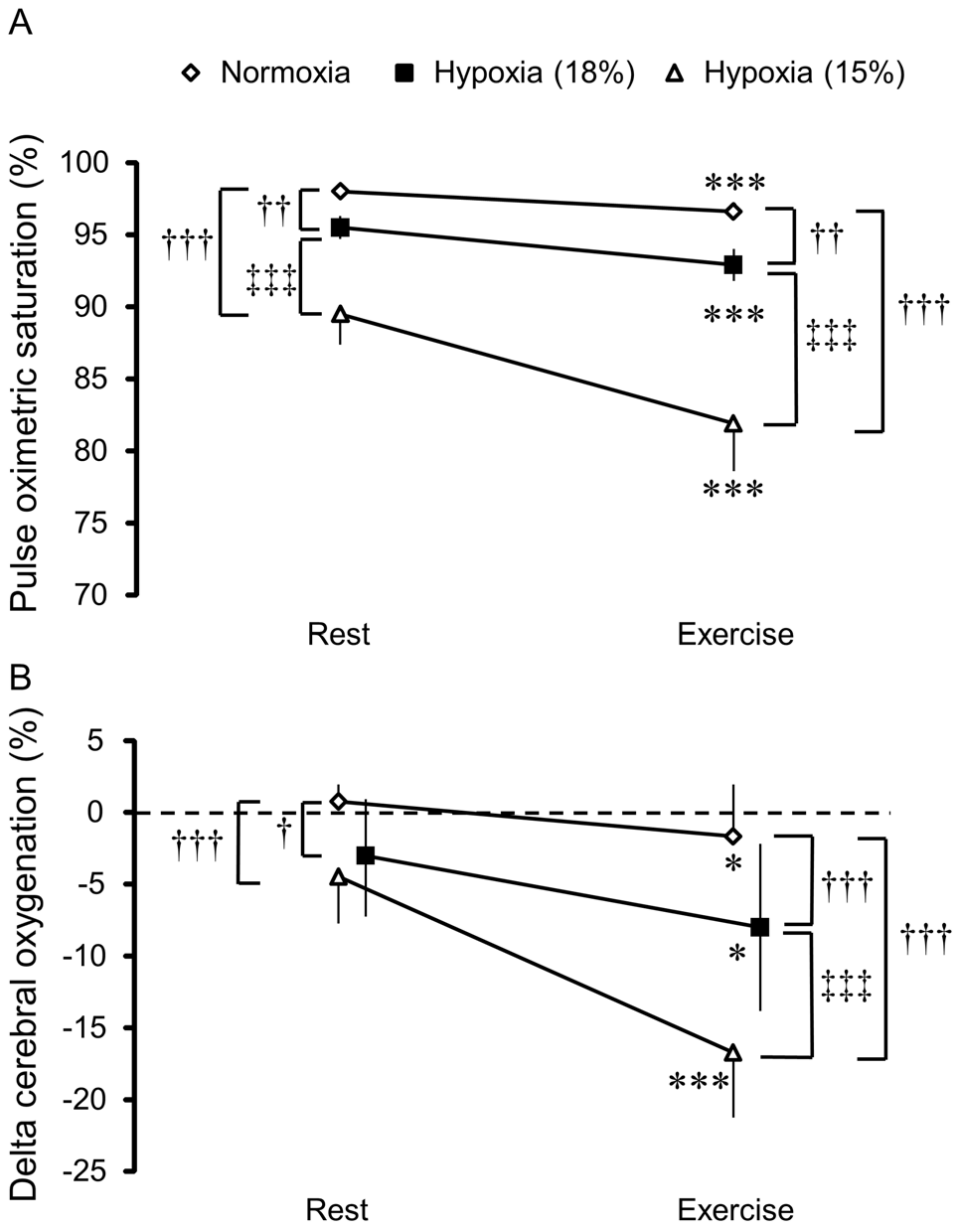
SpO_2_ and cerebral oxygenation during the cognitive task. (A) SpO_2_. (B) Cerebral oxygenation. ^†^P<0.05, ^††^P<0.01, ^†††^P<0.001, vs. Normoxia; ^‡‡‡^P<0.001, vs. Hypoxia at 18% O_2_; *P<0.05, ***P<0.001, vs. Rest.

SpO_2_ gradually decreased at rest and during exercise as the FIO_2_ level decreased (Figure 2*A*). There was a significant interaction between FIO_2_ level and exercise [F(1.30,14.31) = 43.29, *P*<0.001]. This indicates that the degree of decrease in SpO_2_ was greater during exercise as the FIO_2_ level decreased. We observed significant main effects of FIO_2_ level [F(1.19,13.09) = 190.84, *P*<0.001] and exercise [F(1,11) = 102.80, *P*<0.001]. SpO_2_ was lower at rest and during exercise under hypoxia at 18% O_2_ compared to normoxia (*P*<0.01, respectively). Similarly, SpO_2_ was lower at rest and during exercise under hypoxia at 15% O_2_ compared with normoxia and hypoxia at 18% O_2_ (*P*<0.001, respectively). We found significant decreases in SpO_2_ during exercise relative to rest in all FIO_2_ conditions (*P*<0.001, respectively).

Cerebral oxygenation progressively decreased at rest and during exercise as the FIO_2_ level decreased (Figure 2*B*). We obtained a significant interaction between FIO_2_ level and exercise [F(2,22) = 26.01, *P*<0.001], indicating that cerebral oxygenation decreased to a greater degree during exercise as the FIO_2_ level decreased. The main effects of FIO_2_ level [F(2,22) = 63.63, *P*<0.001] and exercise [F(1,11) = 18.38, *P*<0.01] were significant. Cerebral oxygenation was lower at rest (*P*<0.05) and during exercise (*P*<0.001) under hypoxia at 18% O_2_ compared with normoxia. Cerebral oxygenation was lower at rest and during exercise under hypoxia at 15% O_2_ compared with normoxia (P<0.001, respectively). We also found a significant difference in cerebral oxygenation during exercise between hypoxia at 18% and 15% O_2_ (*P*<0.001). Cerebral oxygenation decreased during exercise relative to rest under normoxia (*P*<0.05), hypoxia at 18% O_2_ (*P*<0.05), and hypoxia at 15% O_2_ (*P*<0.001). Collectively, SpO_2_ and cerebral oxygenation decreased in response to exercise and hypoxia in a similar manner. These results suggest that oxygen availability progressively decreased during exercise as the FIO_2_ level decreased.

**Figure 3:**
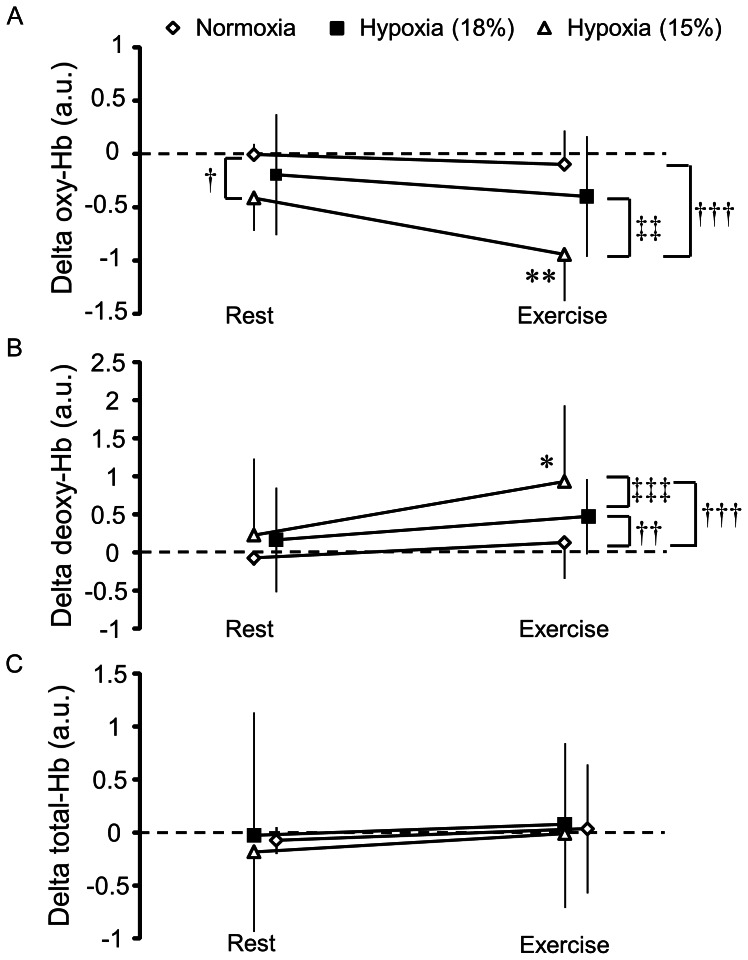
Oxy-Hb, deoxy-Hb, and total-Hb during the cognitive task. (A) Oxy-Hb. (B) Deoxy-Hb. (C) Total-Hb. ^†^P<0.05, ^††^P<0.01, ^†††^P<0.001, vs. Normoxia; ^‡‡^P<0.01, ^‡‡‡^P<0.001, vs. Hypoxia at 18% O_2_; *P<0.05, **P<0.01, vs. Rest.

Figure 3 shows oxy-Hb, deoxy-Hb, and total-Hb concentration changes at rest and during exercise. There was a significant interaction on oxy-Hb between FIO_2_ level and exercise [F(2,22) = 4.81, *P*<0.05]. The main effects of FIO_2_ level [F(2,22) = 16.98, *P*<0.001] and exercise [F(1,11) = 7.23, *P*<0.05] were also significant. We observed a decrease in oxy-Hb at rest under hypoxia at 15% O_2_ compared with normoxia (*P*<0.05). Under hypoxia at 15% O_2_, oxy-Hb significantly decreased during exercise relative to rest (*P*<0.01). As a result, oxy-Hb was lower during exercise under hypoxia at 15% O_2_ compared with normoxia (*P*<0.001) and hypoxia at 18% O_2_ (*P*<0.01).

We observed a significant interaction on deoxy-Hb between FIO_2_ level and exercise [F(2,22) = 6.66, *P*<0.01]. There were significant main effects of FIO_2_ level [F(2,22) = 10.51, *P*<0.01] and exercise [F(1,11) = 5.58, *P*<0.05]. We found an increase in deoxy-Hb during exercise relative to rest under hypoxia at 15% O_2_ (*P*<0.05). Hence, deoxy-Hb was greater during exercise under hypoxia at 15% O_2_ compared with normoxia (*P*<0.001) and at 18% O_2_ (*P*<0.001). In addition, deoxy-Hb was greater during exercise under hypoxia at 18% O_2_ compared with normoxia (*P*<0.01). Total-Hb did not significantly change during the cognitive task in the present study. Overall, we observed a decrease in oxy-Hb and a reciprocal increase in deoxy-Hb during exercise under hypoxia. These results suggest that the decrease in cerebral oxygenation during exercise under hypoxia can be attributed to a decrease in oxy-Hb and an increase in deoxy-Hb.

### Cognitive Task

**Figure 4:**
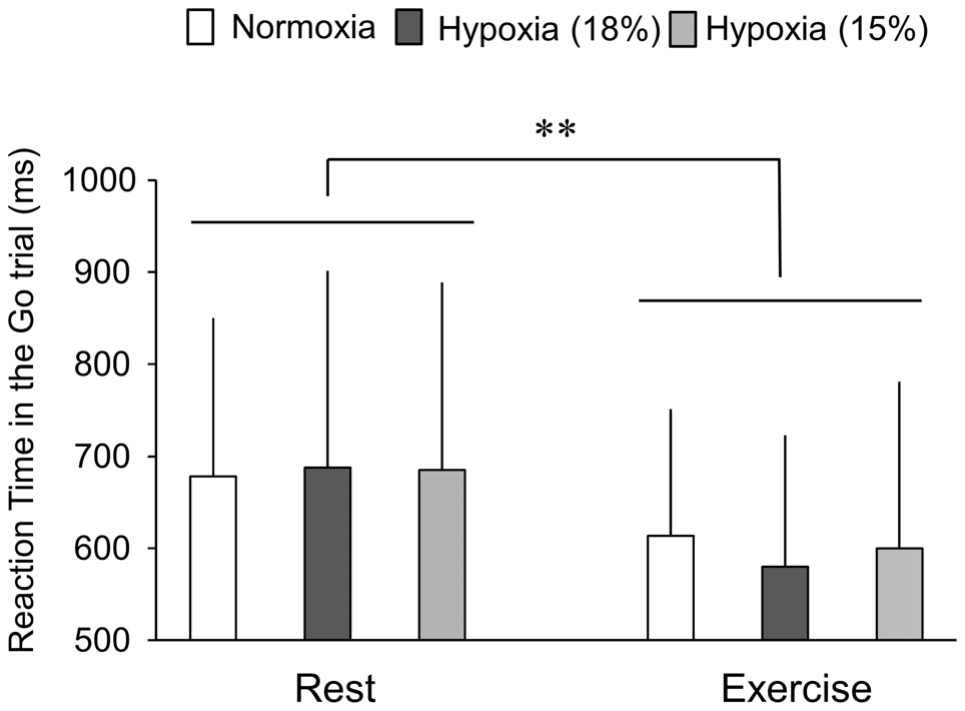
Reaction times in the Go-trial at rest and during exercise under normoxia and hypoxia at 18% and 15% O_2_. **P<0.01, vs. Rest.

Figure 4 shows reaction times in the Go trials. Reaction times decreased during exercise relative to rest [F(1,11) = 18.73, *P*<0.01], whereas differences in FIO_2_ level did not affect reaction times [F(2,22) = 0.06, *P* = 0.94]. There was no significant interaction between FIO_2_ level and exercise [F(1.41,15.53) = 0.06, *P* = 0.69]. These results indicate that reaction time decreased during exercise, and that hypoxia did not affect cognitive function. Table 2 shows response accuracy in the cognitive tasks. Neither differences in FIO_2_ level [F(2,22) = 2.14, *P* = 0.14] nor exercise [F(1,11) = 1.49, *P* = 0.25] significantly affected the response accuracy. These results revealed that the decrease in reaction time was not related to a speed-accuracy trade-off [41]. In the additional experiment, we found no significant differences in reaction times in the first (636±140 ms) and second (674±133 ms) cognitive tasks (P = 0.38). We found no differences in response accuracy between the first (96.8±2.8%) and second (96.5±3.3%) cognitive tasks (P = 0.58). These results suggest that cognitive function was not affected in the additional experiment.

**Table 2:** Response accuracy (%) in the cognitive task under normoxia, hypoxia at 18% O2, and hypoxia at 15% O_2_.

Condition	Response accuracy, %	
	Rest	Exercise
Normoxia	98.1±2.8	95.4±4.1
Hypoxia (18%)	95.3±5.7	94.6±5.3
Hypoxia (15%)	96.9±5.0	97.1±4.9

Values are mean ± SD.

## Discussion

The current study addressed the effects of exercise under hypoxia on cognitive function. We observed that cognitive function improved during a single bout of exercise, consistent with the previous findings. The major findings of this study were: (1) the improvement in cognitive function is due to exercise; (2) surprisingly, in contrast to our initial hypothesis, hypoxia had no effects on cognitive function at least under the present experimental condition.

### Cognitive Function at Rest under Hypoxia

A growing number of studies support the idea that regular aerobic exercise can improve a number of aspects of cognition [42], [43]. These findings demonstrate that high aerobic capacity is beneficial to cognitive function. Hence, oxygen availability may be one of the factors that affect cognitive function at the resting condition. It has been suggested that hypoxia has the potential to impair human brain function [22]. Several previous studies have reported that hypoxia impairs cognitive function in the resting state [23], [24]. In particular, more pronounced impairments were observed under severe hypoxic conditions [23], [24]. In the present study, at the beginning of the experiment of each day, we measured cognitive function at rest under normoxia and hypoxia after exposure. SpO_2_ and cerebral oxygenation significantly decreased at rest under hypoxia compared with normoxia. However, we observed no significant differences in reaction time or response accuracy at rest among the different FIO_2_ conditions. These results may suggest that hypoxic condition was not sufficient enough to impair cognitive function at rest in the present study.

### Cognitive Function during Exercise under Hypoxia

Cognitive function seems to improve during a single bout of moderate exercise [1], [2]. In contrast, decrease in oxygen availability during exercise under hypoxia may impair cognitive function. However, the extent to which exercise under hypoxia affects cognitive function is currently unclear. In the present study, SpO_2_ and cerebral oxygenation progressively decreased during exercise as the FIO_2_ level decreased, suggesting that oxygen availability might be compromised during exercise under hypoxia. This also confirms that our manipulation of available oxygen was successful. In the present study, we observed that reaction time decreased during exercise. However, in contrast to our hypothesis, hypoxia did not affect reaction time. The response accuracy was not affected by exercise or the difference in FIO_2_ level. These results indicate that acute exercise improves cognitive function, and that hypoxia exerted no effects on cognitive function during exercise under the present experimental condition.

It is widely accepted that the exercise-cognition interaction is complex [2], [44], and that the type of cognitive task is an important factor affecting experimental results [44], [45]. In the present study, participants performed the Go/No-Go task, in which they distinguished figures and decided to release or hold a button. Furthermore, participants were asked to find correct responses after the relationship between correct response and figure was reversed or the new figure was presented. This is a cognitive task requiring executive control, including selective attention, response inhibition and interference control [46], [47]. Thus, the present findings suggest that acute exercise improved cognition during exercise. Exercise is reported to influence brain circuits involving neurotransmitters such as dopamine, noradrenaline, serotonin, adrenocorticotropic hormone and cortisol [2], [3], [48], [49]. Some of these physiological changes may be plausible candidates for improvements in cognitive function [1], [2], [50] although the precise mechanisms are yet to be determined. The present study suggests that hypoxia can be effectively ruled out as explanatory variables for performance enhancement benefits derived from acute exercise. Future studies are required to elucidate the mechanisms underlying the improvement in cognitive function during exercise.

Endurance performance is known to decrease under hypoxia compared with normoxia [5]. Recent studies have demonstrated that prefrontal cortex oxygenation [29], [31], [51]–[53] and/or frontal cortex oxygen delivery [54] are limiting factors in maximal exercise performance under severe hypoxia, while a decrease in frontal cortex oxygenation is unlikely to limit exercise performance under normoxia. A study by Amann et al. demonstrated that peripheral fatigue predominantly limits endurance exercise performance under normoxia to moderate hypoxia, whereas central nervous system hypoxia limits endurance exercise performance under severe hypoxic conditions [51]. The authors proposed that the major determinants of endurance exercise performance may switch from peripheral origin of fatigue to a hypoxia-sensitive central component of fatigue below a level of acutely compromised O_2_ transport represented by a range of 70–75% arterial oxygen saturation [51]. This suggests that the effects of exercise under hypoxia on the central nervous system are closely related to the severity of hypoxia. In the present study, the FIO_2_ level of 0.15 was equivalent to moderate hypoxia, and the decrease in SpO_2_ level was above this level during exercise (81.9±3.3%). Moreover, recent studies have shown that severe hypoxia substantially reduced cortical voluntary activation during repeated muscle contraction, while the reduction was less under normoxia to moderate hypoxia [55], [56]. Overall, although cognitive function is not directly comparable to endurance exercise performance and cortical voluntary activation during muscle contraction, it is possible that the severity of hypoxia was not sufficient enough to impair cognitive function under physiological stress. Further research is required to demonstrate whether cognitive function is impaired during exercise under severe hypoxia. In addition, improvements in cognitive function may disappear during prolonged exercise [57]. It also remains unclear whether endurance or strenuous exercise under hypoxia impairs cognitive function.

It could be argued that the improvements in cognitive function we observed might have been due to learning effects. Several previous studies examined cognitive function using control conditions in which cognitive tasks were performed at rest or during exercise at a very low intensity for the same time course [33], [47], [57]–[59]. These studies confirmed that cognitive function was not affected in the control conditions. In the present study, the additional experiment has indicated that cognitive function was not altered without exercise, which is in line with the notion that the improvement in cognitive function is attributable to acute exercise. Furthermore, the participants completed practice blocks before the main experiments, and performed the cognitive tasks under different FIO_2_ conditions in a randomized order. Thus, we can assume that the improvements in cognitive function were due to physiological changes induced by acute exercise. Instead, practicing cognitive tasks improves cognitive performance, which is accompanied by decreased activation in brain areas associated with the task [60], [61]. This process may reflect the shift from controlled to automatic processing [61]. It is possible that practicing the cognitive task enabled more efficient performance and helped minimize the detrimental effects of exercise under hypoxia.

### Limitations

In the present study, we used the same absolute workload in all FIO_2_ conditions, potentially limiting the results. Since HR, RPE, and blood lactate were slightly higher during exercise under moderate hypoxia compared with normoxia and hypoxia at 18% O_2_, it is likely that relative workload was higher during exercise under moderate hypoxia. However, despite relatively high workload during exercise under moderate hypoxia, we observed that acute exercise improved cognitive function under normoxia and hypoxia. Hence, we can assume that using the same absolute workload did not affect our overall conclusion that the improvement in cognitive function is due to exercise, and that hypoxia did not affect cognitive function at least in the present study.

In the present study, we did not include classical control condition. However, we measured cognitive function at rest under normoxia and hypoxia after 10 min exposure. We observed no differences in cognitive performance among different environmental conditions at rest. These results indicate that acute exposure to hypoxia did not affect cognitive function at rest. Thus, we can at least say that acute exposure to hypoxia has negligible effects on cognitive function under the present experimental condition. We are aware that future studies are required to elucidate how cognitive function is affected after exposure to hypoxia for relatively long duration.

Finally, several limitations inherent to NIRS measurement should be noted. First, we measured cerebral oxygenation over the prefrontal cortex during exercise. We cannot detect regional differences in cerebral oxygenation during exercise. Distribution of blood flow to the head and brain is not uniform during exercise [62], [63]. Cerebral blood flow is heterogeneously distributed under hypoxia [64], [65]. Moreover, dynamic cerebral autoregulation may be impaired at rest [66] and during exercise [67] under moderate hypoxia, although hypocapnia is thought to counteract impaired dynamic cerebral autoregulation under hypoxia [68]. As such, the degree of decrease in cerebral oxygenation might differ between brain regions, even though overall patterns of decrease in cerebral oxygenation during strenuous exercise were similar in the prefrontal, premotor, and motor cortices [29]. Second, although we sought to minimize the effects of near-surface blood flow, we cannot rule out the possibility that near-surface blood flow affected cerebral oxygenation measured by NIRS. Third, slight variations in probe placement have been reported to affect cerebral oxygenation [29]. To counteract this possibility, we were careful to place the probe holder at the same position on each experimental day. Previous studies have reported good reproducibility of NIRS measurements during exercise [31], [69]. Finally, cerebral oxygenation reflects the balance between oxygen availability and utilization [70]. This implies that decreases in cerebral oxygenation are not exclusively caused by a decrease in oxygen availability. The observed decrease in cerebral oxygenation during exercise may be, at least in part, due to the increase in oxygen consumption. Despite these shortcomings, NIRS allows the measurement of cerebral oxygenation qualitatively during exercise in a non-invasive manner. We believe that measurement of cerebral oxygenation using NIRS does not compromise the validity of the present findings, which revealed that cognitive function improved during exercise under moderate hypoxia as well as normoxia.

### Conclusions

We examined whether acute exercise under hypoxia affects human cognitive function. We observed that cognitive function improved during a single bout of exercise under normoxia and hypoxia. The present results suggest that the improvement in cognitive function is attributable to exercise, and that hypoxia has no effects on cognitive function at least under the present experimental condition. However, the present results may be closely related to the task demands and/or experimental conditions. In future studies, the exercise-cognition interaction should be examined under various environmental and exercise conditions.
